# An Inquiry-Based Distance Learning Tool for Medical Students Under Lockdown (“COVID-19 Rounds”): Cross-Sectional Study

**DOI:** 10.2196/40264

**Published:** 2023-11-06

**Authors:** Aya Akhras, Mariam ElSaban, Varshini Tamil Selvan, Shaika Zain Alzaabi, Abiola Senok, Nabil Zary, Samuel B Ho

**Affiliations:** 1 College of Medicine Mohammed Bin Rashid University of Medicine and Health Sciences Dubai United Arab Emirates; 2 Department of Medicine Mediclinic City Hospital Dubai Healthcare City Dubai United Arab Emirates

**Keywords:** medical education, COVID-19, technology-enhanced learning, distance learning, student engagement, 5E instructional model

## Abstract

**Background:**

The COVID-19 pandemic presented significant challenges to both clinical practice and the delivery of medical education. Educators and learners implemented novel techniques, including distance learning and web-based rounds, while trying to stay updated with the surge of information regarding COVID-19 epidemiology, pathogenesis, and treatment. Hence, we designed and implemented a technologically enhanced course called “COVID-19 Rounds” to educate students about the rapidly evolving pandemic.

**Objective:**

The objectives of this study are to describe a technologically enhanced course called “COVID-19 Rounds” and evaluate the following: (1) student satisfaction and program usefulness in achieving preset objectives, (2) perceived improvement in literacy regarding the pandemic, and (3) the impact of student engagement by designing infographics and initiating COVID-19–related research projects.

**Methods:**

This is a cross-sectional study measuring the impact of the implementation of the web-based “COVID-19 Rounds” course. This program included web-based clinical experiences with physicians on actual rounds in COVID-19 wards in the hospital, weekly updates on evolving data and new research, and engagement in student-led projects. The study population included 47 fourth-year medical students at the Mohamed Bin Rashid University of Medicine and Health Sciences in Dubai, the United Arab Emirates, who attended the course. We designed and administered a 47-item survey to assess student satisfaction, program usefulness, impact on knowledge, and student engagement. Data were collected at the end of program delivery via Microsoft Forms.

**Results:**

In total, 38 (81%) out of 47 fourth-year medical students participated in this study. The final course evaluation revealed an overall high satisfaction rate, with a mean rating of 3.9 (SD 0.94) on the 5-point Likert scale. Most students were satisfied with the course format (27/38, 71%), organization (31/38, 82%), and the learning experience (28/38, 74%) that the course offered. The course was particularly appreciated for offering evidence-based talks about aspects of the pandemic (34/38, 90%), providing weekly updates regarding emerging evidence (32/38, 84%), and enhancing understanding of the challenges of the pandemic (34/38, 90%). Satisfaction with distance learning was moderate (23/37, 62%), and a minority of students would have preferred an in-person version of the course (10/37, 27%). Student engagement in the course was high. All students participated in small group presentations of infographics of pandemic-related topics. Perceived advantages included conciseness and visual appeal, and disadvantages included the lack of detail and the time-consuming nature of infographic design, especially for students with no prior design experience. After the course ended, 27 (57%) students began research projects. This resulted in 6 abstracts presented at local meetings and 8 scientific papers published or submitted for publication.

**Conclusions:**

This inquiry-based adaptive approach to educating medical students about updates on COVID-19 via web-based learning was successful in achieving objectives and encouraging engagement in research. However, shortcomings of the course related to the lack of in-person teaching and clinical activities were also highlighted.

## Introduction

A key component of a competent physician’s practice is staying up to date with emerging data in their field of expertise and applying that knowledge to clinical practice for the benefit of patients [1]. COVID-19 has highlighted the significance of adapting medical education to ensure that students and physicians can keep up with emerging data and guidelines amid such turbulent times. The surge of data has caused an “infodemic” for health care providers at the front lines [2]. It has also posed a challenge to medical schools, especially for students in their clinical years, with most sites halting their clinical rotations and some experimenting with web-based alternatives [3-5]. The need to continue training physicians in a safe and impactful manner is greater now than ever before [6].

Medical schools have responded to the challenges posed by the COVID-19 pandemic in transformative and innovative ways [7-10]. The Mohammed Bin Rashid University of Medicine and Health Sciences (MBRU) is a newly established medical school in Dubai, located in the health care hub of the United Arab Emirates. The undergraduate medical program at MBRU is based on a competency-based educational model and takes 6 years to complete. The curriculum is divided into 3 phases, with each phase building on the preceding one, resulting in a spiral-like approach. Phase 1 takes place over 1 academic year and covers basic sciences. Phase 2 spans 2 academic years and covers organ systems. Phase 3 spreads over the final 3 academic years and covers clinical sciences. The longitudinal themes curriculum spans the duration of phase 3 and integrates topics such as quality improvement, safe prescribing, and procedural skills.

In response to COVID-19, the MBRU phase 3 clinical sciences faculty introduced “COVID-19 Rounds” as part of the longitudinal themes curriculum to provide physicians and patients with web-based clinical experiences in hospital COVID-19 wards. The goal of the course was to actively engage students with the unfolding challenges of a new pandemic. Learning theories were incorporated including the biopsychosocial model of illness and the 5E (Engage, Explore, Explain, Elaborate, and Evaluate) model framework for learning, initially described by the Biological Sciences Curriculum Study for science teaching and adapted to medical education [11,12].

Based on this model, essential components of the course included web-based clinical experiences with COVID-19, weekly updates on local and global epidemiological statistics, information about microbiology and transmission, and emerging evidence about diagnosis and treatment. Students presented COVID-19–related infographics and research projects, with the aim of contributing to medical knowledge by participating in scientific conferences and publishing in peer-reviewed journals. Our goal for this study is to report on the experience with this inquiry-based web learning course for medical students in their clinical years during the pandemic.

This study aims to (1) describe a technology-enhanced course named “COVID-19 Rounds” to educate medical students regarding a rapidly developing pandemic and (2) evaluate the course in terms of student satisfaction and program usefulness in meeting preset objectives, perceived improvement in literacy regarding the COVID-19 pandemic, and the impact of student engagement (by designing infographics and initiating research projects).

## Methods

### Study Participants

We conducted a cross-sectional study and collected data via a survey at 1 time point at the end of program delivery. The study participants included 38 (out of 47 who enrolled in the course) fourth-year (out of 6 years) Bachelor of Medicine and Bachelor of Surgery medical students at MBRU. All students in the fourth-year class were part of our sample, as this “COVID-19 Rounds” course was delivered under the longitudinal theme’s curriculum at MBRU. This was an optional pass/fail course with the requirements of a student assignment. There was a 100% pass rate observed. The course ran from March 29, 2020, to September 1, 2020, with a total of 22 sessions delivered via Microsoft Teams (Microsoft Corp). This course was delivered while student clinical rotations were converted to web-based learning and continued through the summer.

### Curriculum Development

The “COVID-19 Rounds” curriculum was designed by the university’s clinical faculty, taking into consideration the rapidly evolving nature of information about Sars-CoV-2 and the COVID-19 pandemic. The Engel biopsychosocial model of instruction [13,14] and inquiry-based learning via the 5E learning framework [15] were utilized to inform and enhance the curriculum. The biopsychosocial model is strongly applicable to the COVID-19 pandemic due to the complex interaction of biological mechanisms of disease; population factors related to disease control; and the social, political, and psychological elements related to vaccinations and group dynamics [14]. Students were encouraged to reflect on the pandemic’s evolution from a wider lens and consider the various biological, psychological, and social aspects at play. The 5E learning framework is a type of inquiry-based learning approach to allow students to construct learning experientially. This was implemented by engaging students through the stages of engagement, exploration, explanation, elaboration, and evaluation [15]. The development was also informed by carrying out interactive activities and discussions and incorporating different levels of instruction ascending the Bloom taxonomy of learning from basic didactic instruction to self-directed group tasks [16].

### Data Collection

Quantitative and qualitative data were collected by open survey method utilizing Microsoft Forms and created based on the objectives of the study (Multimedia Appendix 1). Data collection was open for 10 weeks, starting at the end of program delivery to allow for maximum response rates and accounting for factors that could influence response rates, such as coinciding exams. Convenience sampling was used based on the context of the study and was made available to the 47 fourth-year medical students who took the course. The survey was sent via email, along with a description of the study (Multimedia Appendix 1) and a link to input responses into Microsoft Forms.

The survey consisted of 4 sections and 47 questions spanning 9 pages. It required 7 minutes to complete on average. The sections were entitled “Satisfaction with COVID-19 Rounds,” “COVID-19 Rounds Effectiveness,” “Impact of “COVID-19 Rounds on Knowledge and Level of Understanding,” and “Added Value of the Web-Based Program and Use of Infographics.” The initial survey draft was pilot-tested to establish face validity, correct poorly-worded questions and unambiguity, and shorten it to reduce respondent fatigue. Most of the data were measured via a 5-point Likert scale [17] ranging from strongly disagree to strongly agree. The course rating was via a 5-star rating scale. In total, 6 open-ended questions were included to assess expectations, perceived advantages and disadvantages associated with web-based course delivery, and infographic design. Responses to open-ended questions were analyzed by quantifying the responses, and they were given descriptively. Different answers that were variations of the same meaning were grouped by mutual agreement of the investigators.

### Ethics Approval

This study was approved by the MBRU Institutional Review Board (MBRU-IRB-2020-26). Participation was completely voluntary. The participants were provided with a written consent document and were asked to agree to the study; if so, they were automatically sent the link to the survey. Participant data remained anonymous and completely deidentified, and privacy and confidentiality protections were guaranteed. Participants could discontinue at any time during the process of data collection and skip any sensitive questions as needed. No compensation was provided for participation in this study. Moreover, participants were able to review and change their answers if needed and request a summary of their responses at the end of the survey. There was no automated way to prevent multiple entries, but there were no known or apparent incentives for this to occur. Data were stored in an encrypted file on a secure computer.

### Statistical Analysis

Stata 16 software (StataCorp) and Microsoft Excel were used for statistical analyses. All 47 fourth-year medical students were eligible to participate in the study. Power calculations, based on a CI of 95%, yielded a target sample size of 42 medical students. Frequencies with proportions were reported for categorical variables, including the questions graded via the Likert scale. Additionally, means with SDs were reported for continuous variables.

## Results

### Course Description

The “COVID-19 Rounds” course consisted of a guided inquiry-based approach to web-based learning based on the 5E instructional model of 5 phases to engage, explore, explain, elaborate, and evaluate [11]. The program was presented online via the Microsoft Teams platform for 1 hour twice weekly to the whole fourth-year class. The presentations were divided into several sections. First, the students were engaged by physicians who were videotaped in actual rounds in COVID-19 wards, presenting patients along with laboratory and radiologic data. This enabled students to experience the events unfolding in hospitals in real time despite the remoteness of the lockdown. Second, the program promoted exploration by providing frequent updates on the status of the pandemic in terms of the epidemiology of the infection in various countries. Following the Engel biopsychosocial model of disease, the updates included information both on scientific developments related to the virology of COVID-19 and the societal and economic effects of the pandemic. Third, students were engaged in explaining by being divided into groups and tasked to design and present infographics about COVID-19, spanning topics such as epidemiology, microbiology, diagnosis, treatment, immunology, and the psychosocial/ economic impact of the COVID-19 pandemic (Multimedia Appendix 2). Next, the students were encouraged to elaborate by building on these topics and emergent knowledge by working in groups to develop novel research questions and lead research projects in collaboration with faculty and clinicians. Finally, the students were able to evaluate their knowledge via a postcourse questionnaire, as well as in the revision and review processes inherent in the publication experience. This model has been shown to be effective in enhancing student progress in science-related instruction [11].

### Student Satisfaction and Program Usefulness

**Figure 1:**
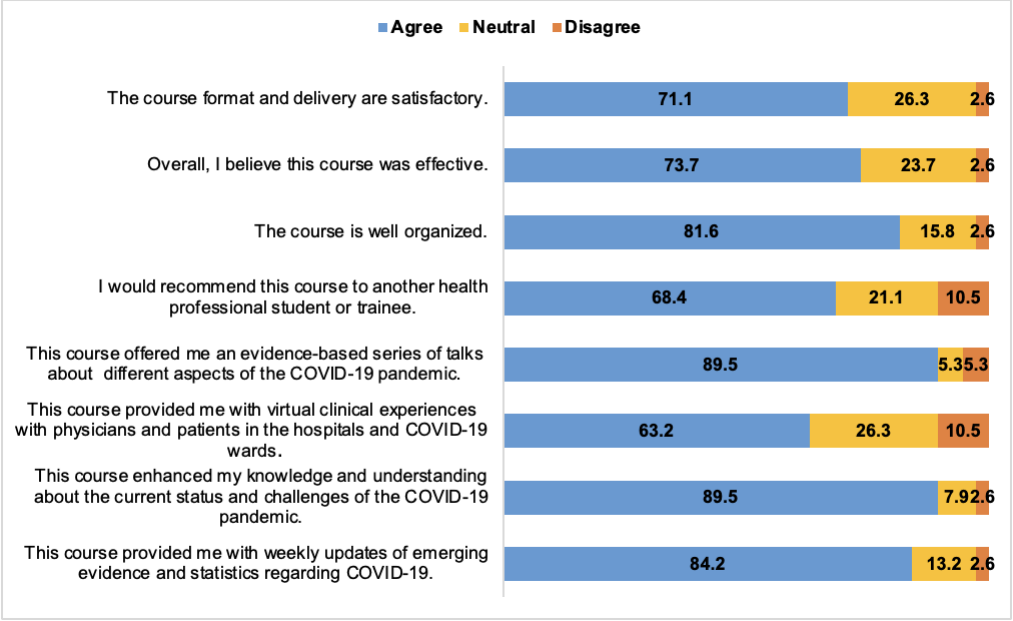
Survey responses: student satisfaction and program effectiveness.

A total of 38 (81%) fourth-year medical students participated in this study, out of the 47 students who participated in the course. Overall, student satisfaction was high, with a mean rating of 3.9 (SD 0.94) on a Likert scale from 1 (low satisfaction) to 5 (high satisfaction). Overall, most students agreed that the course format and delivery were satisfactory (27/38, 71%), effective (28/38, 74%), and well-organized (31/38, 82%). In addition, 62% (23/37) of the students agreed that the distance learning component of the course was beneficial, while 27% (10/37) would have preferred a face-to-face version of the course. Almost half of the students (18/37, 49%) indicated that the web-based format stimulated interest in participating. Figure 1 provides further details about the student satisfaction rates measured via the survey.

The preset objectives included providing weekly evidenced-based talks, enhancing understanding of the status and challenges of the pandemic, providing web-based clinical experiences with patients, and encouraging student-led research. The course was successful in meeting the preset objectives. Overall, 90% (34/38) of the students agreed that the course offered an evidence-based series of talks regarding different aspects of the COVID-19 pandemic. However, the value of the web-based clinical experience was not uniformly agreed upon. Only 63% (24/38) of students agreed that the course provided a satisfactory web-based clinical experience with patients in hospitals and COVID-19 wards. Further data regarding meeting preset objectives can be found in Figure 1.

### Perceived Improvement in Literacy About the COVID-19 Pandemic

We investigated students’ improvement regarding the pandemic literacy or knowledge base. Our data indicate that the course increased most of the students' knowledge about COVID-19 (27/38, 72%) and was appropriate for the students’ level of understanding (31/37, 84%). Importantly, the course increased the large majority (32/38, 84%) of the students’ confidence in understanding COVID-19 data.

Additionally, 92% (35/38) of the students agreed that keeping up with emerging data is important. Despite this belief, more than half (20/38, 53%) of the students admitted to facing challenges with keeping up to date with emerging evidence regarding COVID-19 prior to the course. It is important to highlight the students’ engagement with other information resources outside of this curriculum as well. Before the course, only 53% (20/38) of the students reported not being able to keep up with emerging data and evidence. Moreover, despite finding the course helpful, less than half (16/38, 42%) relied solely on the COVID-19 rounds to keep up to date.

**Figure 2:**
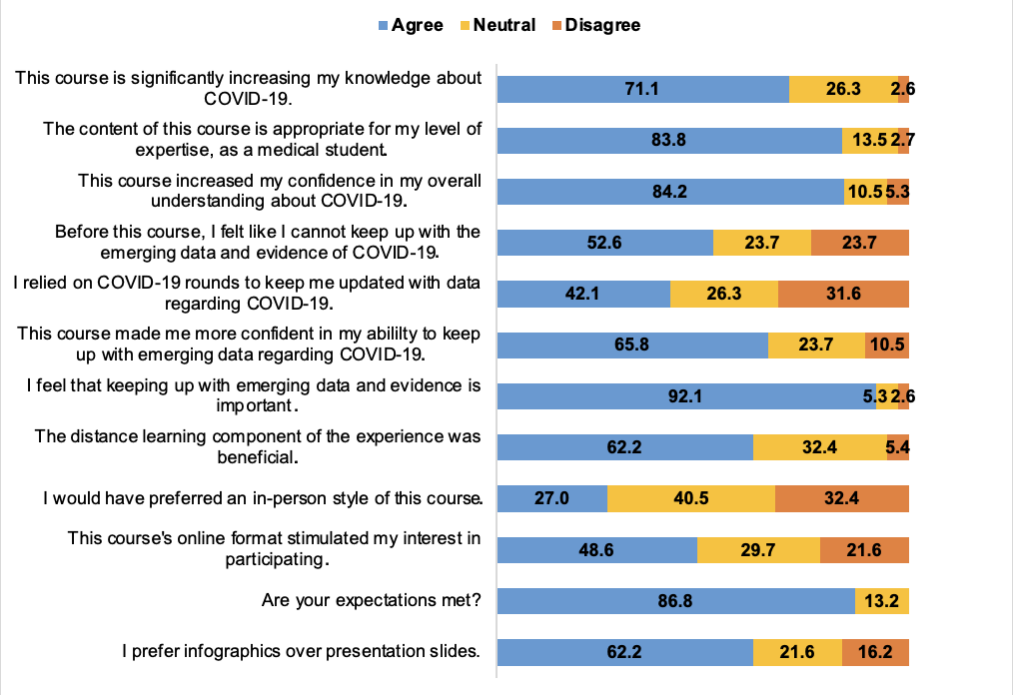
Survey responses: perceived improvement in literacy regarding the COVID-19 pandemic.

Furthermore, the course enhanced students’ confidence in teaching others about various aspects of COVID-19, such as local and international guidelines (28/38, 74%), immunology and vaccination development (26/38, 68%), and the psychosocial and economic impact of the pandemic (26/38, 68%). The course allowed 58% (22/38) of the students to feel confident in understanding statistics after COVID-19 rounds. Questions related to student literacy and the development of the infographics can be found in Figure 2. These data show that students agreed that the course increased knowledge, expertise, knowledge, and confidence and met expectations.

### Impact of Designing Infographics and Initiating Research

We sought to investigate the course’s impact on student engagement through the process of creating infographics and initiating research projects. Overall, 74% (28/38) of the students had designed infographics before taking this course. Many students liked using infographics to present data (28/37, 76%) and appreciate that infographics are becoming more popular in the medical literature (28/37, 6%). Students also felt that infographics are a valuable tool for presenting and summarizing information (32/37, 86%). In total, 62% (22/37) preferred infographics over presentation slides.

Furthermore, one of the long-term goals of the course was to promote student research. As illustrated in Table 1, during the course, 27 (57%) students developed and worked on 10 research projects. Most have been published in peer-reviewed journals to date and have contributed to the development of important student skills.

**Table 1:** Student-led COVID-19 research projects.

Project	Student participants^a^, n (%)	Status
“Telehealth to the Rescue During COVID-19: A Convergent Mixed Methods Study Investigating Patients’ Perceptions”	5 (11)	Abstract presented; paper published [18]
“Spectrum of Disease Of COVID-19 Among Hospitalized Adults in the UAE^b^”	7 (15)	Abstract submitted to local meeting
“Clinical Characteristics of Children With COVID-19: A Cross-Sectional Multi-Center Study in the UAE”	4 (9)	Abstract presented; paper published [19]
“COVID-19 Under 19: A Meta- Analysis”	4 (9)	Paper published [20]
COVID-19 and Health Care Workers: A Systematic Review and Meta-Analysis”	5 (11)	Abstract presented; paper published [21]
“The COVID-19 Pandemic and Health and Care Workers: Findings From a Systematic Review and Meta-Analysis (Second Wave) 2021-2022”	5 (11)	Paper published [22]
“Risk factors Related to an Outbreak of COVID-19 Among Health Care Workers in a General Medicine Hospital Ward”	4 (9)	Abstract presented [23]; paper submitted
“Clinical and Epidemiological Features and Severity Markers in Children Admitted With Multisystem Inflammatory Syndrome in Children (MISC)”	4 (9)	Paper submitted [24]
“COVID-19 Rounds”: An Inquiry-Based Distance Learning Tool for Medical Students Under Lockdown: A Cross-Sectional Study”	3 (6)	Abstract presented [25]; (current paper)
“Persistent Symptoms Following Recovery from COVID-19”	7 (15)	Abstract presented [26]; Study ongoing

^a^Individual students and faculty may have been involved in more than 1 project.

^b^UAE: United Arab Emirates.

### Expectations, Advantages, and Disadvantages of COVID-19 Rounds

At the end of the questionnaire, we included a component consisting of open-ended questions to provide students with the opportunity to express their thoughts and expand on the reasoning behind their choice of questionnaire options. These questions pertained to expectations of COVID-19 rounds; perceived advantages and disadvantages of the course; and advantages, disadvantages, and challenges of and feedback on designing infographics. The classification of students’ responses can be found in Table 2.

As indicated in Table 2, part of assessing the preset objectives included asking students about their expectations of the course and providing an opportunity for feedback in cases where their expectations were not met (5/38, 13%). In general, most respondents were expecting updates on the COVID-19 pandemic, spanning domains such as statistics, management, treatment, and research. Regarding unmet expectations, some students felt the course was “repetitive” and “more like a journal club.”

Most respondents agreed that an advantage of the course was providing regular updates on the status of the pandemic and being easily accessible over the internet (Table 2). The disadvantages voiced pertained to the timing of the course in relation to exams/other courses, being didactic in nature, decreased interaction due to the course delivery format, and technical difficulties during patient rounds. Students expressed that the web-based nature of the course was disadvantageous in some ways due to decreased interaction and challenges with communication. Some students indicated that attending the talks could be time-consuming prior to exams or that the talks were occasionally “more didactic than true rounds.”

The perceived advantages and disadvantages of infographics were explored (Table 2). The stated advantages commonly revolved around the course’s simplicity, conciseness, and visual appeal (Multimedia Appendix 2). On the other hand, the common perceived disadvantages of infographics include a lack of detail, course information becoming outdated quickly, the time-consuming design, and the fact that it required design experience rather than traditional PowerPoint skills. Additionally, 1 student spoke about the disadvantage of the inability to incorporate multimedia into infographics.

The common challenges experienced by students include issues with teamwork over the internet, formatting, challenges with condensing information into small spaces, and meeting course deadlines.

**Table 2:** Classification of the students’ responses.

Expectations of COVID-19 rounds			Occurrences of the theme, n (%)
**Category**			
	Updates^a^		34 (89)
	Scientific knowledge		1 (3)
	Personal protective equipment		1 (3)
	Management of COVID-19		1 (3)
**Feedback for unmet expectations**			
	Information not useful		2 (5)
	Didactic		1 (3)
	Little patient interaction		1 (3)
	Repetitive		1 (3)
**Advantages and disadvantages of COVID-19 rounds**			
	**Advantages**		
		Updates	16 (42)
		Accessibility	5 (13)
		Social Distancing	2 (5)
	**Disadvantages**		
		Difficulty with web-based communication	4 (11)
		Timing (scheduling issues, class schedules)	4 (11)
		Less patient interaction	4 (11)
		Technical difficulties	1 (3)
		Didactic	1 (3)
**Infographics: advantages, disadvantages, and challenges faced**			
	**Advantages**		
		Concise and easy to understand	16 (42)
		Visually appealing	8 (21)
		Accessible	4 (11)
		Teamwork	3 (8)
	**Disadvantages**		
		Lack of detail	7 (18)
		Time-consuming to design	4 (11)
		Require design experience	4 (11)
		Condensed information is hard to understand	2 (5)
		Quickly outdated	1 (3)
		Difficult to present online	1 (3)
		Inability to incorporate multimedia	1 (3)
	**Challenges**		
		Requires design experience	9 (24)
		Lack of teamwork	6 (16)
		Summarizing	5 (13)
		Time management and meeting deadlines	5 (13)
		Formatting	4 (11)
		Literature search	2 (5)
		Lack of objectives	1 (3)
		Difficulty with web-based team communication	1 (3)

^a^Some students indicated that they were expecting updates about management (n=6, 6%), statistics (n=5, 13%), research (n=2, 5%), local implications (n=1, 3%), and global implications (n=1, 3%).

## Discussion

### Principal Results

This inquiry-based learning approach to educating medical students about COVID-19 was successful in achieving high student satisfaction rates, educating students about the pandemic during lockdown, and promoting active student engagement in research. The format of this course resulted in high levels of student engagement in explaining through designing infographics and elaborating and evaluating by engaging in original research. In addition, students expressed appreciation for the rising importance of infographics in medical literature, but they also recognized the limitations of this format. The shortcomings of the course related to challenges with the lack of in-person teaching and actual clinical activities with COVID-19 patients. Overall, we hope that this initial experience can help bridge the literature gap regarding adaptive and inquiry-based educational strategies implemented amid the COVID-19 pandemic.

Due to the limitations of an almost universal lockdown, classroom teaching and clinical rotations in the hospital were limited to web-based instructional strategies. Similar to all medical schools worldwide, we adopted a new web-based teaching experience that had not been specifically described before the onset of the pandemic. We used known software and tools such as Microsoft Teams, in addition to videotaping physicians who were working on COVID-19 care wards, coupled with presenting emerging new data by field experts. Students were engaged using an inquiry approach that involved both explanation and exploration, with new emerging data involving the assignment of creating infographics to explain new information to other students, followed by exploration through new research ideas. We found similar challenges to what has been described [10], including challenges related to technological failures faced by both faculty clinicians and students during the web-based rounds on the COVID-19 wards and trying to maintain proper infection control measures while using video-calling technology. Additional challenges included engaging students in new forms of design and difficulties modifying these formats with continually changing data. We have previously documented the major challenges faced by the faculty during this time, specifically in dealing with uncertainties, unfamiliar web-based teaching and meeting programs, and blurring of work-life balance [8,10]. These findings informed subsequent web-based learning efforts at our institution.

Students expanded on their expectations, perceived advantages, and disadvantages of the course. Our results indicated that students appreciated regular updates on the pandemic due course’s ease of access via the internet; however, they expressed that some components of the course were more didactic in nature and that they experienced scheduling conflicts and technical difficulties with web-based ward rounds. The feedback indicated that more active engagement in learning rather than didactics was appreciated, as emphasized by the students’ enthusiasm about infographic presentations and their widespread involvement in research. Overall, students appreciated the conciseness and visual appeal of the course, but they expressed difficulties related to the lack of detail, the design experience requirements, and the fact that this format may not be suitable for rapidly emerging data, which are prone to becoming outdated quickly.

The feedback from this experience shed light on potential improvements for the clinical distance learning climate at our institution. Going forward, we will acknowledge the importance of providing updates balanced with student engagement and participation to avoid didactic instructional styles. Moreover, we will focus on improving the technology used in wards to avoid any difficulties. Finally, we will implement the use of infographics for topics less prone to continuous changes and provide short tutorials on infographic design prior to student assignments.

### Comparison With Prior Work

The learning challenges experienced with COVID-19 have been recently summarized. Systematic reviews have detailed the numerous distance learning strategies that medical schools developed during COVID-19, including technology-enhanced learning and technology-based clinical education, such as web-based rounds, bedside teaching, and clinic visits [7,27]. A recent systematic review of 51 publications related to remote learning in response to the COVID-19 pandemic described multiple remote learning strategies, including moving classrooms to synchronous activities with various web-based engagement techniques. They found that evaluations were generally positive regarding attitudes and increasing knowledge and skills [10].

In addition, the challenges faced by medical school faculty and educators in dealing with uncertainty, rapid decisions, and lack of experiential learning have been outlined [8]. Student attitudes included higher satisfaction rates with distance learning if they had prior experience with it and when instructors actively participated in their sessions using multimedia and active engagement [28]. They also appreciated the flexibility of web-based learning [29]. A student survey in the United Kingdom found that students greatly increased their online learning during COVID-19 with a perceived advantage of flexibility but struggled with family distractions and internet connectivity issues [29].

After our experience, several other academic centers published their experience specifically related to clinical online teaching. Hofmann et al [30] reported a similar adaptational experience by implementing web-based rounds via Zoom [31]. In total, 92% of the students in that study strongly agreed that this experience improved their literacy about the pandemic, which is similar to our study’s results. Overall, they had a favorable student response but faced similar challenges with audio and network issues.

Additionally, medical schools worldwide have reported on their experiences with web-based rounds spanning many subspecialty clerkships [32-34]. Muhammad et al [35] conducted a systematic review of 201 studies reporting on the effectiveness of web-based medical teaching during the COVID-19 pandemic. Overall, many studies reported positives of web-based teaching, including flexibility, convenience, engagement, effectiveness as an alternative to in-class teaching, and increased networking with worldwide specialists. In our study, students preferred the convenience of web-based learning and the ability to keep up with emerging data. Common disadvantages of web-based teaching observed by Mohammad et al [35] included the loss of face-to-face interaction and decreased engagement [36-41]. Sud et al [42] and Michael et al [43] postulate that the loss of direct student-teacher interaction could be a factor for decreased student engagement. Khamees et al [10] reported similar challenges, including a lack of hands-on learning and social interaction and frustrations with technology. These disadvantages are similar to those in our study, despite the overall course being rated as useful.

While many universities have had to redesign clinical curricula during the lockdown, our study aims to highlight a key cornerstone of future physicians rising through this pandemic and becoming actively engaged at multiple levels using an inquiry-based approach. A cornerstone of the “COVID-19 Rounds” course is its engagement of students in their learning process. This was accomplished by the inquiry-based format and active learning by generating infographics and engaging in student-led research projects with clinicians and faculty. Students were instructed to use infographics as the mode of presentation rather than traditional PowerPoint presentations. Most of our students were familiar with infographics and appreciated their aesthetically pleasing, concise nature and rising importance in medical literature. A frequently voiced opinion was the challenge of condensing the breadth of information into a summarized format, as well as the technical and design skills required to design them compared to the traditional presentation. From the British Medical Journal to the Journal of the American Medical Association, infographics have served as a concise, yet eye-catching way to visually present information. Infographics allow readers to receive a message more quickly and effectively than a paragraph with the same content [44,45].

Engaging students in the learning process involved giving them opportunities to design and lead original research with faculty members. The majority of students actively participated in a total of 8 research projects, resulting in 6 abstract presentations at local scientific research conferences. To date, all 8 research projects have been published in peer-reviewed and indexed journals. This was facilitated by the engagement of senior clinician-researchers with these student-led projects. The students were able to leverage the cancelation of clinical placements and spend that time doing research. Studies on the impact of adapting to distance learning during clinical training should consider opportunities for new types of learning, such as those highlighted in this paper.

Additionally, MBRU adopted similar strategies to this course’s models in their online teaching, including learning principles and the ability to motivate students to initiate research projects. This focus on research projects as an application has been applied to other courses in the College of Medicine at MBRU, including a 2-year longitudinal course on quality improvement and health systems.

### Limitations

Our study has its limitations. First, the small sample size limits its generalizability. This could be mitigated by administering the course to more student years (1-6) or other medical schools. Second, technical challenges in course delivery due to confidentiality limited student interaction with patients and clinical education. This could be mitigated in the future by using a secure encrypted portal for student education to allow for more interaction with patients over the internet. Third, we did not have precourse data to compare pre- and postcourse knowledge improvement, resulting in a descriptive study. In the future, pre- versus postcourse literacy could be better calculated via *t* tests and regression models to help elucidate gaps in the course, as well as its effectiveness. However, the salient strengths of our study include that this was one of the first in the literature reporting on the implementation of a web-based learning platform with an inquiry-based focus on educating medical students about COVID-19. It also served as a platform for discussions and participation to gather high-quality evidence and engage in research presentations and publications. Our study provides insight into the advantages of providing students with a structured method for weekly updates on emerging evidence and their receptiveness to engagement and participation in learning.

### Conclusions

Our inquiry-based and active learning approach with online technology via the “COVID-19 Rounds” course has helped foster lifelong learners in the age of the “infodemic.” Our weekly web-based course, which provided medical students with updates about COVID-19, increased pandemic knowledge and literacy and encouraged peer-to-peer education and engagement in research. However, shortcomings of the course related to the lack of in-person teaching and clinical opportunities were also highlighted.

## Appendix

Multimedia Appendix 1 Postcourse survey material for students.

Multimedia Appendix 2 Examples of infographics designed by students.

Multimedia Appendix 3 "COVID-19 Rounds" data set.

## Abbreviations

5E: Engage, Explore, Explain, Elaborate, and Evaluate
MBRU: Mohamed Bin Rashid University of Medicine and Health Sciences

## Data Availability

The data sets generated and/or analyzed during the study are available in Multimedia Appendix 3.
