# Supplementary material for: An Inquiry-Based Distance Learning Tool for Medical Students Under Lockdown (“COVID-19 Rounds”): Cross-Sectional Study
Source: JMIR Med Educ. 2023 Nov 6;9:e40264. doi: 10.2196/40264 (PMC10629505; doi:10.2196/40264)
Supplement: Multimedia Appendix 1 [file mededu_v9i1e40264_app1.pdf]

Investigating the impact of virtual “COVID-19 Rounds” during the COVID-19 Pandemic

Dear Student,

The purpose of this prospective cross-sectional study is to investigate the impact "COVID-19 Rounds" as an innovative educational program during COVID-19 through measuring student satisfaction, the effectiveness of the program, impact on the level of knowledge and understanding, and exploring the added value of infographics to the learning experience.

In response to COVID-19, Mohammed Bin Rashid University for Medicine and Health Sciences (MBRU) introduced several initiatives to increase the adaptability and resilience of their students. Among those initiatives is “COVID-19 Rounds” which is meant to be a contextualized, curriculum-based program that aims to provide virtual clinical experiences with physicians and patients in the hospitals and COVID-19 wards, to educate students with an evidence-based series of talks.

There will be no identifying information linked to any responses. Your participation in this study is voluntary and anonymous. You may choose not to participate. There are no perceived risks from participating in this study. You can discontinue at any time during the process of data collection and can skip any sensitive questions when needed. Your participation will also help us understand how to improve teaching related to helping students regarding these issues in the future. The results of this study will be used for educational purposes and potentially be disseminated for publication or presented at conferences.

Please contact the study investigators if you have any questions
Aya Akhras Aya.akhras@students.mbru.ac.ae (mailto:Aya.akhras@students.mbru.ac.ae)

* Required

Consent

Your participation in this study is voluntary and anonymous. Your information will be confidential and stored in a securely encrypted file for use with regards to the purpose of this study only.

1. By clicking proceed, you understand the above information and are willingly participating in this survey. *

   ◯ Proceed

Satisfaction with "COVID-19 Rounds"

2. Please rate the following statements: *

	Strongly Disagree	Disagree	Neutral	Agree	Strongly agree
I feel that attending and participating in COVID-19 Rounds is a good use of my time.	○	○	○	○	○
The course format and delivery are satisfactory.	○	○	○	○	○
The course is well organized.	○	○	○	○	○
The learning experience offered to me as part of the COVID-19 Rounds is satisfactory.	○	○	○	○	○
I would recommend this course to another health professional student or trainee.	○	○	○	○	○

"COVID-19 Rounds" Effectiveness

3. What were you expecting to learn as a part of COVID-19 Rounds?

4. Are your expectations met?

- ◯ Yes
- ◯ No

5. If your expectations are not met, please elaborate:

6. Please rate the following statements:

	Strongly Disagree	Disagree	Neutral	Agree	Strongly agree
This course provided me with virtual clinical experiences with physicians and patients in the hospitals and COVID-19 wards	○	○	○	○	○
This course offered me an evidence-based series of talks about different aspects of the COVID-19 pandemic.	○	○	○	○	○
This course enhanced my knowledge and understanding about the current status and challenges of the COVID-19 pandemic.	○	○	○	○	○
This course provided me with e a weekly update of emerging evidence and statistics regarding COVID-19.	○	○	○	○	○
Overall, I believe this course was effective.	○	○	○	○	○

Impact of "COVID-19 Rounds" on Knowledge and Level of Understanding

7. Please rate the following statements:

	Strongly Disagree	Disagree	Neutral	Agree	Strongly agree
This course is significantly increasing my knowledge about COVID-19.	○	○	○	○	○
The content of this course is appropriate for my level of expertise, as a medical student.	○	○	○	○	○
This course increased my confidence in my overall understanding about COVID-19.	○	○	○	○	○
This course increased my confidence to teach others about COVID-19 Epidemiology.	○	○	○	○	○
This course increased my confidence to teach others about COVID-19 Diagnosis.	○	○	○	○	○
This course increased my confidence to teach others the local and international guidelines and protocols around COVID-19.	○	○	○	○	○
This course increased my confidence to teach others about COVID-19's Immunology and Vaccine Status	○	○	○	○	○
This course increased my confidence to teach others about COVID-19's Psychosocial and Economic Impact.	○	○	○	○	○

	Strongly Disagree	Disagree	Neutral	Agree	Strongly agree
I am comfortable educating others about COVID-19 as a result of this course	○	○	○	○	○
I feel a sense of responsibility to educate my family and friends about COVID-19, with knowledge gained from this course	○	○	○	○	○
Before this course, I felt like I cannot keep up with the emerging data and evidence of COVID-19	○	○	○	○	○
I relied on COVID-19 rounds to keep me updated with data regarding COVID-19	○	○	○	○	○
This course made me more confident in my abililty to keep up with emerging data regarding COVID-19	○	○	○	○	○
I feel that keeping up with emerging data and evidence is important	○	○	○	○	○
I feel confident understanding statistics after the COVID-19 rounds	○	○	○	○	○

8. After this course, I see myself constantly keeping up-to-date with emerging data and evidence in my future career

0	1	2	3	4	5	6	7	8	9	10

Not at all likely   Extremely likely

Added Value of the Virtual Program and Use of Infographics

9. Please rate the following statements:

	Strongly Disagree	Disagree	Neutral	Agree	Strongly agree
The distance learning component of the experience was beneficial.	◯	◯	◯	◯	◯
The course allowed me and my colleagues to express ourselves creatively when creating infographics.	◯	◯	◯	◯	◯
I like the use of infographics as a mode of presenting data.	◯	◯	◯	◯	◯
I enjoyed creating infographics and presenting them.	◯	◯	◯	◯	◯
I prefer infographics over presentation slides.	◯	◯	◯	◯	◯
I believe infographics are a valuable tool for presenting and summarizing information	◯	◯	◯	◯	◯
I think infographics are becoming more popular in medical literature	◯	◯	◯	◯	◯
I would have preferred an in-person style of this course	◯	◯	◯	◯	◯
This course's online format stimulated my interest in participating	◯	◯	◯	◯	◯
This course's use of infographics stimulated my interest in participating	◯	◯	◯	◯	◯

	Strongly Disagree	Disagree	Neutral	Agree	Strongly agree
I am more likely to design infographics in the future	○	○	○	○	○

10. Have you designed infographics before "COVID-19 Rounds"?

○ Yes

○ No

11. What challenges did you encounter while designing the infographics?

12. What are the advantages/ disadvantages of designing infographics for data presentation?

13. What were the advantages/disadvantages of having virtual COVID-19 rounds?

14. Any comments regarding the course or infographics?

15. I would like to see this course repeated for other topics

0	1	2	3	4	5	6	7	8	9	10

Not at all likely Extremely likely

16. I would rate this course as:

☆ ☆ ☆ ☆ ☆

This content is neither created nor endorsed by Microsoft. The data you submit will be sent to the form owner.

Microsoft Forms
