# Supplementary material for: An Inquiry-Based Distance Learning Tool for Medical Students Under Lockdown (“COVID-19 Rounds”): Cross-Sectional Study
Source: JMIR Med Educ. 2023 Nov 6;9:e40264. doi: 10.2196/40264 (PMC10629505; doi:10.2196/40264)

B. Examples of COVID-19 infographics

COVID-19 Epidemiology – 07/04/2020

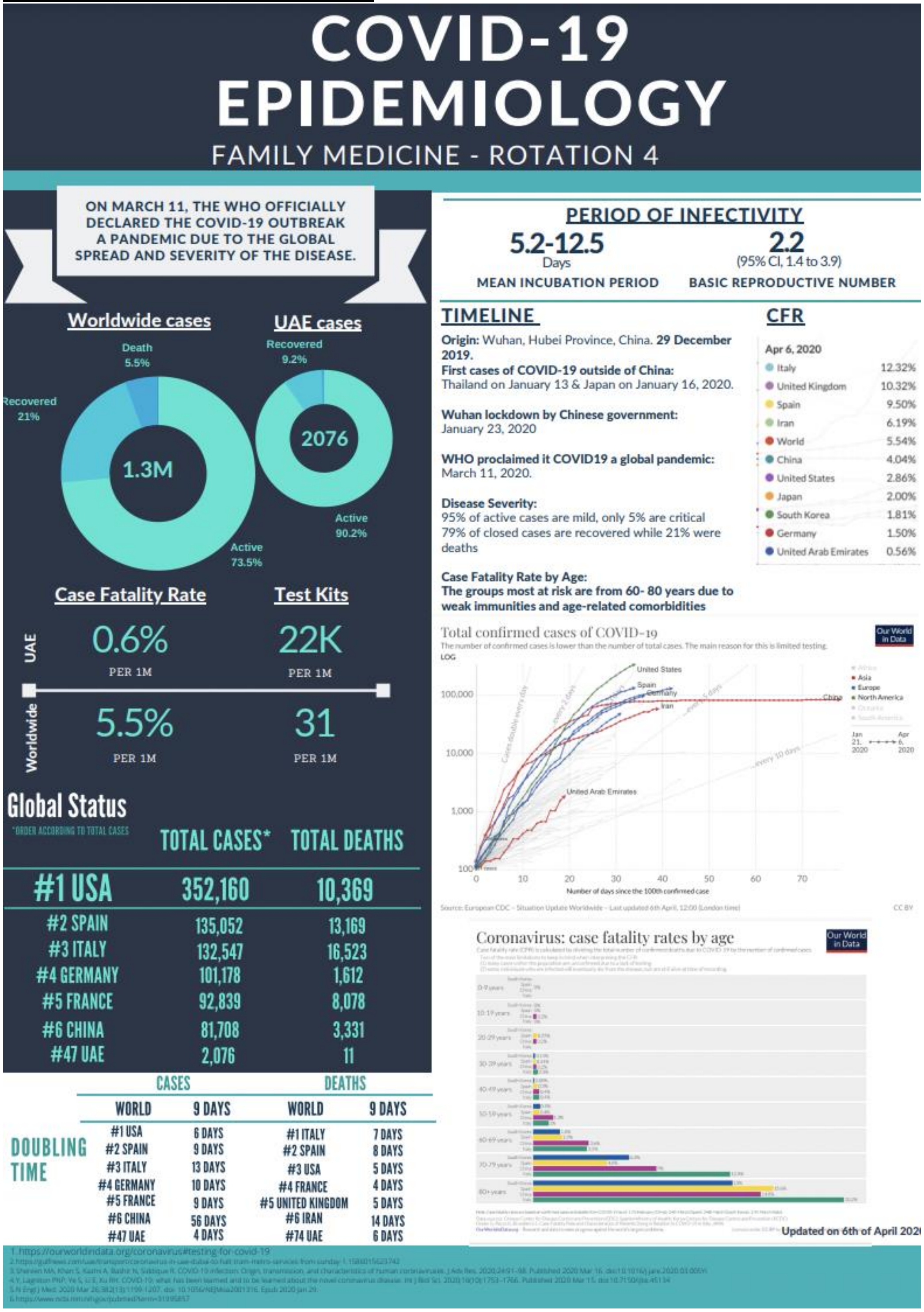

COVID-19 Diagnosis – 12/04/2020

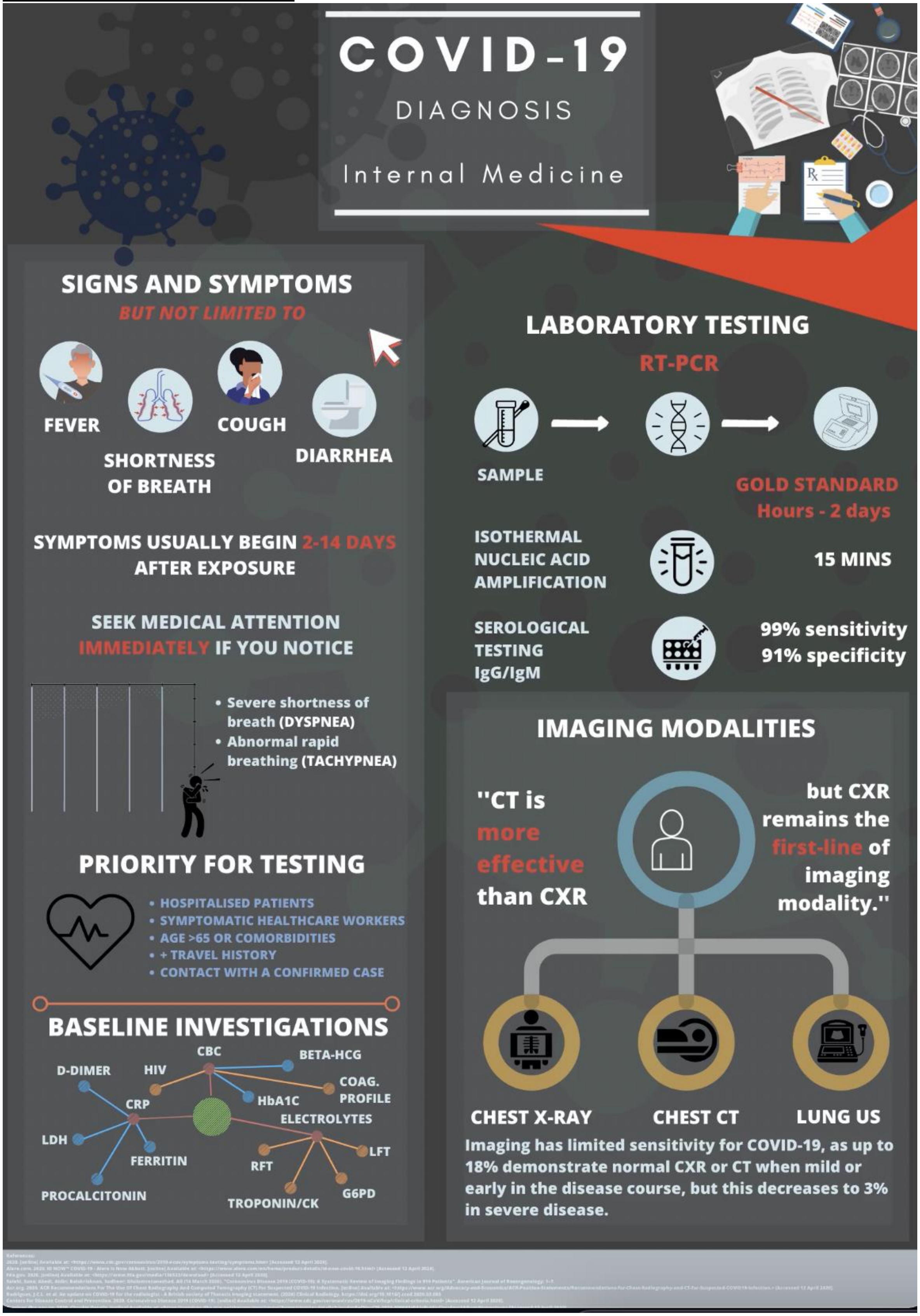

COVID-19 Psycho-social-economic impact – 21/04/2020

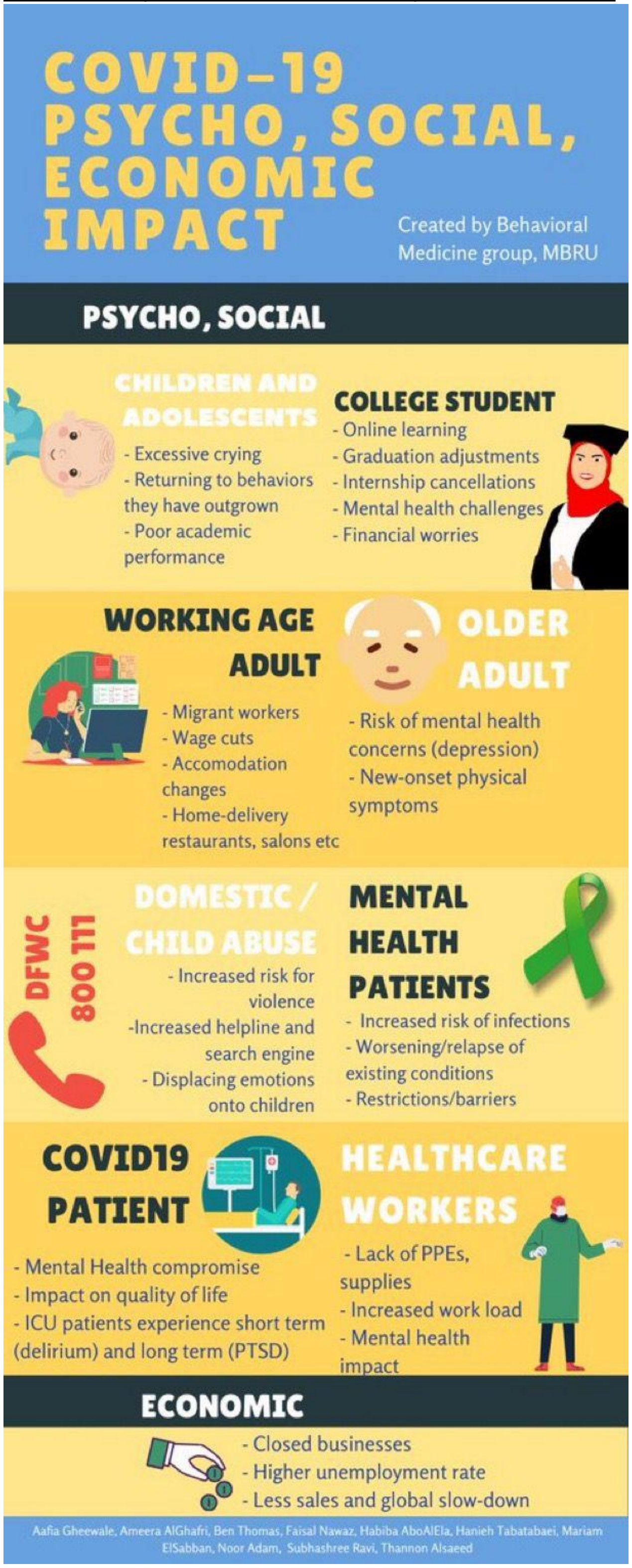

Supplement: Multimedia Appendix 2 [file mededu_v9i1e40264_app2.pdf]
